# Sialyl-Tn vaccine induces antibody-mediated tumour protection in a relevant murine model

**DOI:** 10.1038/sj.bjc.6605083

**Published:** 2009-05-12

**Authors:** S Julien, G Picco, R Sewell, A-S Vercoutter-Edouart, M Tarp, D Miles, H Clausen, J Taylor-Papadimitriou, J M Burchell

**Affiliations:** 1 Breast Cancer Biology Group, King's College London, London SE1 9RT, UK; 2 Unite de Glycobiologie Structurale et Fonctionnelle, UMR USTL/CNRS no 8576, IFR 147, Université des Sciences et Technologies de Lille, Villeneuve d'Ascq F-59655, France; 3 Department of Cellular and Molecular Medicine, Faculty of Health Sciences, University of Copenhagen, Copenhagen N, Denmark; 4 Mount Vernon Cancer Centre, Mount Vernon Hospital, Rickmansworth Road, Northwood, Middlesex HA6 2RN, UK

**Keywords:** STn, breast cancer, Theratope, immunotherapy, OPN

## Abstract

Changes in the composition of glycans added to glycoproteins and glycolipids are characteristic of the change to malignancy. Sialyl-Tn (STn) is expressed by 25–30% of breast carcinomas but its expression on normal tissue is highly restricted. Sialyl-Tn is an *O*-linked disaccharide that can be carried on various glycoproteins. One such glycoprotein MUC1 is expressed by the vast majority of breast carcinomas. Both STn and MUC1 have been considered as targets for immunotherapy of breast cancer patients. Here we used different immunogens to target STn in an MUC1 transgenic mouse model of tumour challenge. We show that synthetic STn coupled to keyhole limpet haemocyanin (Theratope), induced antibodies to STn that recognised the glycan carried on a number of glycoproteins and in these mice a significant delay in tumour growth was observed. The protection was dependant on STn being expressed by the tumour and was antibody mediated. Affinity chromatography of the STn-expressing tumour cell line, followed by mass spectrometry, identified osteopontin as a novel STn-carrying glycoprotein which was highly expressed by the tumours. These results suggest that if antibodies can be induced to a number of targets expressed by the tumour cells, a humoral response can be effective in controlling tumour growth.

Carcinogenesis is often accompanied by over-expression and/or aberrant glycosylation of various proteins by cancer cells. For instance, over-expression of MUC1 carrying truncated and sialylated *O*-glycans is considered as a common feature of breast and other carcinomas (Taylor-Papadimitriou *et al*, 2002). This aberrant glycosylation can directly generate cancer-associated antigens in two ways: (1) by exposure of cryptic protein epitopes that are normally masked by the extended glycosylation, (2) by changing the composition of the carbohydrate side chains attached to the protein resulting in, for example, the Thomsen–Friedenreich (T), Thomsen-nouveau (Tn) antigens and their sialylated version sialyl-T (ST) and sialyl-Tn (STn). The loss of polarity of the epithelial cancer cells allows the exposition of these antigens on the whole surface of the cell making them accessible to antibodies and cells of the immune system.

The STn antigen consists of a simple disaccharide expressed in about 30% of breast cancers (Julien and Delannoy, 2003; Miles and Papazisis, 2003). The importance of this glycan is demonstrated by the association of its expression with a decreased overall survival of breast cancer patients (Julien and Delannoy, 2003) and the lack of response to chemotherapy (Miles *et al*, 1994). Expression of STn has been shown to be dependant on the expression of the sialyltransferase ST6GalNAc I in breast cancer cell lines (Julien *et al*, 2001, 2005; Sewell *et al*, 2006) and totally correlated with ST6GalNAc I expression in primary breast tumours (Sewell *et al*, 2006). As the expression of STn is highly restricted in normal tissues (Julien and Delannoy, 2003), this antigen has been considered of interest for the development of anticancer vaccine. A synthetic STn-keyhole limpet haemocyanin (KLH) vaccine (Theratope), which consists of 3000 mol of disaccharide conjugated to 1 mol of KLH, has been designed by the biotech company Biomira (now Oncothyreon, Alberta, Canada; Ragupathi *et al*, 1999). Pre-clinical studies in mice showed that immunisation with Theratope can induce STn-specific IgG (for review see Miles and Papazisis, 2003). Moreover, a phase II clinical study in breast cancer patients showed that potent STn-specific humoral responses can be induced following immunisation with this immunogen, which correlated with the overall survival of patients (Miles and Papazisis, 2003). However, in the follow-up phase III trial no benefit for Theratope-immunised patients compared to control groups was shown. It must be noted however that STn expression was not evaluated as a prerequisite for study entry and therefore the possibility of a benefit in a subset of patients whose tumours expressed STn could not be excluded.

The most common glycoprotein that can carry the STn glycan in breast carcinomas is the membrane-bound mucin known as MUC1. In breast cancer patients, both humoral (Kotera *et al*, 1994; von Mensdorff-Pouilly *et al*, 2000; Snijdewint *et al*, 2001) and cellular (Correa *et al*, 2005) responses to MUC1 have been documented and a number of clinical studies have been initiated to investigate the use of MUC1-based immunogens in different tumour types (Tang *et al*, 2008). Unglycosylated MUC1 peptides corresponding to the tandem repeat domain have been used to vaccinate patients (Tang *et al*, 2008) and immune responses have been noted. However, when humoral responses were seen, the antibodies were unable to recognise glycosylated MUC1, in contrast to naturally occurring antibodies (von Mensdorff-Pouilly *et al*, 2000).

In this study we used a Balb/c mouse model to investigate the efficacy of using STn-carrying immunogens to inhibit tumour growth. For this we developed a tumour cell line (E3STn) that expresses STn and MUC1 by the transfection of MUC1 and ST6GalNAc I, the sialyltransferase responsible for the formation of STn, into 410.4 mammary tumour cell line. MUC1 glycopeptides, MUC1 glycoprotein and STn formulations were used as immunogens in an MUC1 transgenic model before challenging with the STn-expressing tumour cells. We found that Theratope (KLH-STn) vaccination induced tumour protection that was dependant on the expression of STn by the tumour cells and the presence of antibodies that could recognise a number of STn-carrying proteins.

## Materials and methods

### Mice

Balb/c transgenic mice, homozygous for human MUC1 transgene expression, were described previously (Sorensen *et al*, 2006). Balb/c mice deficient for mature B cells and the production of antibodies due to the expression of mutant *μ*MT alleles (Qin *et al*, 1998) were a generous gift from Prof Thomas Blankenstein, Max Delbruck Centre for Molecular Medicine, Berlin. All animal experiments were performed under Home Office project licence PPL70/5930 observing UKCCCR guidelines.

### Cell lines

Mouse mammary carcinoma cell line E3, which expresses human MUC1, has been described previously (Smith *et al*, 1999). Stable transfection of murine ST6GalNAc I was performed to obtain the cell line E3-STn by the method described previously (Sewell *et al*, 2006). Stable transfectants for human ST6GalNAc I have been previously described for MDA-MB-231 (Julien *et al*, 2001) and T47-D (Sewell *et al*, 2006). MCF-7-STn was generated as described (Julien *et al*, 2001, 2005).

### MUC1-derived glycopeptides and glycoprotein

MUC1-pep-unglycosylated consisted of 60 amino acids (60mer) comprising three tandem repeats of MUC1. To obtain MUC1-pep-Tn, the same peptide was glycosylated *in vitro* by recombinant UDP-*N*-acetylgalactosamine (UDP-GalNAc): polypeptide GalNAc-transferases as described (Sorensen *et al*, 2006; Tarp *et al*, 2007). MUC1-pep-Tn is fully *O*-glycosylated with all potential 15 sites for *O*-glycosylation carrying a GalNAc. MUC1-pep-STn was obtained by sialylating MUC1-pep-Tn *in vitro* by recombinant ST6GalNAc I (Sorensen *et al*, 2006). All 15 sites for *O*-glycosylation were carrying an STn disaccharide. MUC1-pep-STn was conjugated to KLH as a carrier molecule before immunisation (Sorensen *et al*, 2006; Tarp *et al*, 2007).

Recombinant MUC1-carrying STn was purified from CHO-K1-expressing ST6GalNAc-1 as described previously (Sewell *et al*, 2006). The density of *O*-glycosylation has been previously investigated and an average of 3.8 of the five potential sites of *O*-glycosylation of each tandem repeat was glycosylated (Sewell *et al*, 2006).

### Theratope immunisation

Three days before the immunisation schedule, mice were given an intravenous (i.v.) injection of 2.3 mg of cyclophosphamide. Theratope immunogen, provided by Biomira, was diluted in PBS and mixed with the enhanzyn adjuvant. Enhanzyn consists of Detox-B, which is an emulsion of monophosphoryl lipid A, mycobacterial cell wall and lecithin (Holmberg and Sandmaier, 2001). Mice were then subcutaneously (s.c.) injected with 10 *μ*g of either Theratope with adjuvant or KLH with adjuvant in a total volume of 200 *μ*l three times at three weekly intervals. Three weeks after the final injection the mice were challenged with 100 *μ*l of 5 × 10^6^ cells per ml E3-STn or E3 tumour cell suspended in PBS by s.c. injection into the flank.

### MUC1-STn immunisation

Three days before the first immunisation, mice were given an i.v. injection of 2.3 mg of cyclophosphamide. Mice were then injected s.c. with 10 *μ*g of MUC1-pep-STn coupled to KLH or with 10 *μ*g of MUC1-prot-STn mixed with Freund's adjuvant (Sigma, Poole, UK) in a total volume of 200 *μ*l. Each animal received a total of four injections at two weekly intervals. Two weeks after the final injection the mice were challenged with 100 *μ*l of 5 × 10^6^ cells per ml E3-STn or E3 tumour cell suspended in PBS by s.c. injection into the flank.

### ELISA

Microtitre plates were coated with antigen overnight at 4°C with ovine submaxillary mucin (OSM; 200 ng per well), MUC1-pep-STn (50 ng per well), MUC1-pep-Tn (50 ng per well) or MUC1-pep-unglycosylated (50 ng per well) and the ELISA was performed as described previously (Tarp *et al*, 2007). Isotypes of antibodies in the sera were determined on OSM plates using class-specific second antibodies.

### Tumour monitoring

Tumour development was monitored three times per week by bi-dimensional measurement using vernier calipers. Tumour volumes in mm^3^ were calculated as (*a* × *b*^2^)/2, where *a* is the largest measure and *b* is the smallest in mm. Mice were killed when tumours reached 1–1.5 cm or if they ulcerated. Tumour-free survival was analysed using Kaplan–Meier model in SPSS software (SPSS UK Ltd., Woking, UK). Each animal was considered to be positive when a measurable tumour (13.5 mm^3^) was reported. Statistical significance was assessed using the Breslow test for which *P*<0.1 is considered significant.

### Immunohistochemical staining of tumour sections

Frozen sections of human primary breast carcinomas, selected for their expression or lack of expression of STn, were fixed in ice-cold methanol/acetone (1 : 1) for 10 min. Sections were stained with mouse serum (diluted 1 : 400) or with the monoclonal antibody (mAb) to STn, TKH2 as described (Sorensen *et al*, 2006).

Formalin-fixed, paraffin-embedded sections or frozen section of E3-STn tumours fixed in 4% paraformaldehyde in PBS (30 min) were stained with the antibodies detailed in the Results followed by an biotinylated-anti-mouse IgG (Dako, Ely, UK) diluted 1 : 200 and StreptABComplex/HRP (Dako) as previously described (Sorensen *et al*, 2006).

### Affinity chromatography

TKH2 was coupled to CNBr-activated Sepharose 4 Fast Flow (GE Healthcare, Amersham, UK). E3-STn cells were lysed in Tris-HCl 50 mM (pH 7.0), NaCl 150 mM, Triton X-100 2% (pH 7) for 4 h on ice and spun to remove large cell debris. Total cell lysate (6 mg protein) was then loaded into the column and the column was washed with 10 column volumes of PBS before elution with 0.1 M glycine at pH 2.5. The fractions were immediately neutralised and submitted to SDS–PAGE and western blotted with TKH2.

### Electrophoresis and western blotting

Cell pellets (5–30 × 10^6^ cells) were lysed in 50 mM Tris-HCl, 150 mM NaCl buffer containing 1% Triton X-100. Proteins (100 *μ*g) or recombinant osteopontin (OPN; 1 *μ*g; R&D, Abingdon, UK) were loaded onto 4–12% gradient acrylamide gel, submitted to SDS–PAGE electrophoresis under reducing conditions and electro-transferred on nitrocellulose membranes in accordance with standard procedures.

Membranes were blocked with 1% BSA in TBS and incubated with primary antibodies: anti-STn TKH2, anti-MUC1 HMFG1, polyclonal goat IgG anti-OPN or anti-sera from immunised mice in TBS Tween 20 0.05%, for 1 h. After washing, labelled proteins were revealed using appropriate secondary antibodies conjugated to alkaline-phosphatase and NBT/X-phosphate revelation reagent (Roche, Lewes, UK).

### Identification of proteins by nano-HPLC-tandem mass spectrometry

Proteins eluted from anti-STn affinity chromatography were lyophilised, dissolved in Laemmli buffer and separated by SDS–PAGE (7.5–10% gradient). The gel was silver-stained and the bands of interest were cut and de-stained in a solution containing 1.6% sodium thiosulfate and 1% potassium ferrycyanate. After reduction and alkylation, proteins were submitted to in-gel trypsin digestion overnight at 37°C in 25 mM ammonium bicarbonate buffer (sequencing grade modified trypsin; Promega, Charbonnieres, France) and extracted tryptic peptides were submitted to nano-HPLC-tandem mass spectrometry analysis as previously described (Gurcel *et al*, 2008). Nano-HPLC-nanoESI-MS/MS analyses were performed on an ion trap mass spectrometer (LCQ Deca XP+; Thermoelectron, San Jose, CA, USA) equipped with a nano-electrospray ion source coupled to a nano flow high-pressure liquid chromatography (HPCL) system (LC Packings Dionex, Amsterdam, the Netherlands).

Fragment ion spectra were searched against the Swiss-Prot *mus musculus* database (SwissProt 55.2, 362782 sequences, 130497792 residues) using the MS/MS ion search Mascot software (Matrix Science, London, UK). The search parameters were 1.5 Da tolerance for the parent ion mass (1+, 2+ or 3+ charged) and 0.8 Da for the MS/MS fragment ions, one missed cleavage allowed, carbamidomethylcysteine as fixed modification and methionine oxidation as possible modification. Protein hits were ranking according to the protein scores derived from ions scores. Only the proteins with a significant score were considered (>35 with Swiss-Prot database).

### Immunoprecipitation

Snap-frozen tumours were crushed using a Mikro-Dismembrator II (Braun Biotech, Melsungen, Germany). Tumour powders were dissolved in lysis buffer containing the Complete Mini anti-protease cocktail (Roche) and incubated for 2 h at 4°C. Lysates were homogenised and further incubated for 2 h. Total tumour lysates (0.5 mg of proteins) were pre-cleared using 50 *μ*l of A/G proteins agarose-beads (Roche) for 2 h and incubated with 2 *μ*g of anti-OPN antibody for 3 h. A/G proteins beads were subsequently added and incubated overnight. Immunoprecipitated proteins were washed using TBS 0.05% Tween 20, the eluted from the bead using elution buffer (0.1 M glycine at pH 2.5) for two incubation of 15 min each. All procedures were carried out at 4°C using ice-cold reagents. The precipitates were then submitted to SDS–PAGE and western blotting as described above.

## Results

### Immunisation with synthetic STn coupled to KLH (Theratope) induced a humoral response specific to STn that was not restricted to MUC1-STn

To investigate the efficacy of STn to induce an immune response, STn conjugated to KLH in the form of Theratope was used to vaccinate both wild-type (WT) Balb/c mice and MUC1 transgenic mice on a Balb/c background. The MUC1 transgenic mice were selected as a model as in breast cancer MUC1 is the major mucin that carries STn. When Theratope was used in the clinical setting, patients were given low-dose cyclophosphamide with the aim of reducing their T-regulatory cells and so enhancing the immune response (Miles and Papazisis, 2003). Thus, both strains of mice received a single i.v. injection of 2.3 mg of cyclophosphamide 3 days before vaccination with Theratope or KLH.

**Figure 1:**
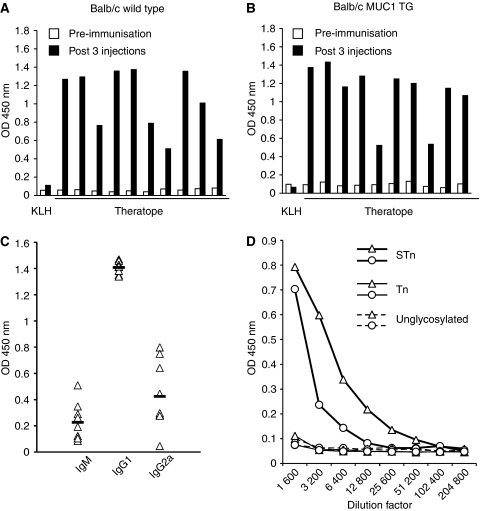
Immunisation with Theratope induces anti-STn antibodies. Wild-type (**A**) or human MUC1 transgenic (MUC1 TG). (**B**) Balb/c mice were immunised with Theratope or KLH alone (negative control). Sera were collected pre- and post-immunisation. Presence of anti-STn-specific antibodies was assessed by ELISA using OSM-coated plate. Each set of bars represents 1 : 3000 diluted serum from a distinct animal (1 representative for the control group and 10 for the test group). (**C**) Isotypes of anti-sera (1 : 3000 dilution) obtained from MUC1 TG after immunisation with Theratope was determined by ELISA using OSM-coated plates and IgM-, IgG1- or IgG2a-specific second antibodies. (**D**) Serial dilution of sera from Balb/c WT (triangle) or Balb/c MUC1 transgenic (circle) immunised with Theratope were analysed by ELISA in wells coated with 50 ng of MUC1-pep-STn, MUC1-pep-Tn or MUC1-pep-unglycosylated. Each curve is representative of three independent sera tested. OD, optical density.

**Figure 2:**
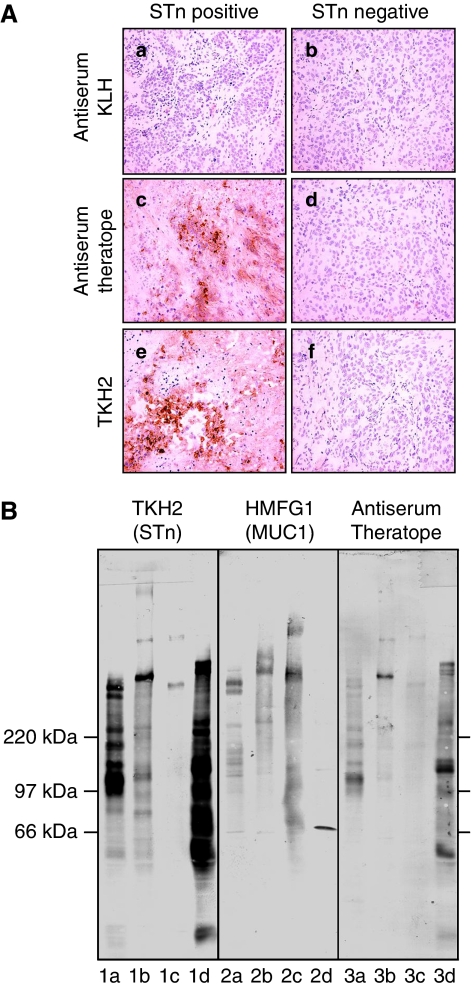
Theratope-induced anti-sera are able recognise STn-expressing breast carcinomas and a broad range of STn-positive proteins. (**A**) Sections of STn-positive (**a**, **c**, **e**) or STn-negative (**b**, **d**, **f**) human breast carcinoma were stained sera from control KLH-immunised mice (**a**, **b**), Theratope-immunised MUC1 TG mice (**c**, **d**) or with TKH2 mAb (**e**, **f**) to verify STn expression. This is representative of three different anti-sera tested. (**B**) E3-STn (**1a**, **2a**, **3a**), MCF-7-STn (**1b**, **2b**, **3b**), T47-D-STn (**1c**, **2c**, **3c**) and MDA-MB-231-STn (**1d**, **2d**, **3d**) were subjected to western blot analysis with TKH2 (STn), HMFG1 (MUC1) or anti-sera from the Theratope-immunised mice as indicated. The figure is representative of three sera tested.

After three injections, anti-STn antibodies were detected by ELISA in all the mice that received Theratope (Figure 1A and B). The antibodies were mostly IgG1 but IgG2a and IgM were also observed (Figure 1C). The anti-sera induced by Theratope immunisation in both WT and MUC1 transgenic mice were able to react with MUC1-pep-STn (60mer with all 15 *O*-linked glycosylation sites carrying STn) but not with MUC1-pep-Tn (60mer with all 15 sites carrying Tn) or MUC1-pep-unglycosylated (60mer) (Figure 1D). Furthermore, the anti-sera were found to react with human primary breast cancers expressing STn but were unable to react with STn-negative tumours (Figure 2A), even though they expressed the Tn antigen (data not shown).

The ability of anti-STn sera raised in MUC1 TG mice to react with various STn-positive proteins was analysed by western blotting. E3-STn and three STn-positive human breast cancer cell lines, MCF-7-STn, MDA-MB-231-STn (Julien *et al*, 2001) and T47-D-STn (Sewell *et al*, 2006), were used as a source of STn-positive proteins. As shown in Figure 2B (left panel), these cell lines expressed variable amounts of different STn-positive proteins detected using the TKH2 mAb. MUC1 (middle panel) is present in E3-STn, MCF-7-STn and T47D-STn but barely detectable in MDA-MB-231-STn cell lysates. Bands corresponding to MUC1 could be recognised by TKH2 in E3STn and the epithelial breast cancer cell lines MCF7-STn and T47-D-STn. Anti-sera from mice immunised with Theratope were weakly able to detect bands of similar mobility to MUC1-STn (right panel lanes 3a, 3b and 3c) but reacted more strongly with other STn proteins expressed in E3-STn or MDA-MB-231-STn cell line. Although the intensity of the staining obtained was weaker than seen with TKH2, the detection of the various STn proteins was found to be qualitatively similar. Moreover, no staining was observed using the same anti-sera to blot cell lysates from the parental cell lines not transfected with ST6GalNAc I and therefore not expressing STn (data not shown).

### Vaccination with Theratope induced tumour protection in Balb/c mice

**Figure 3:**
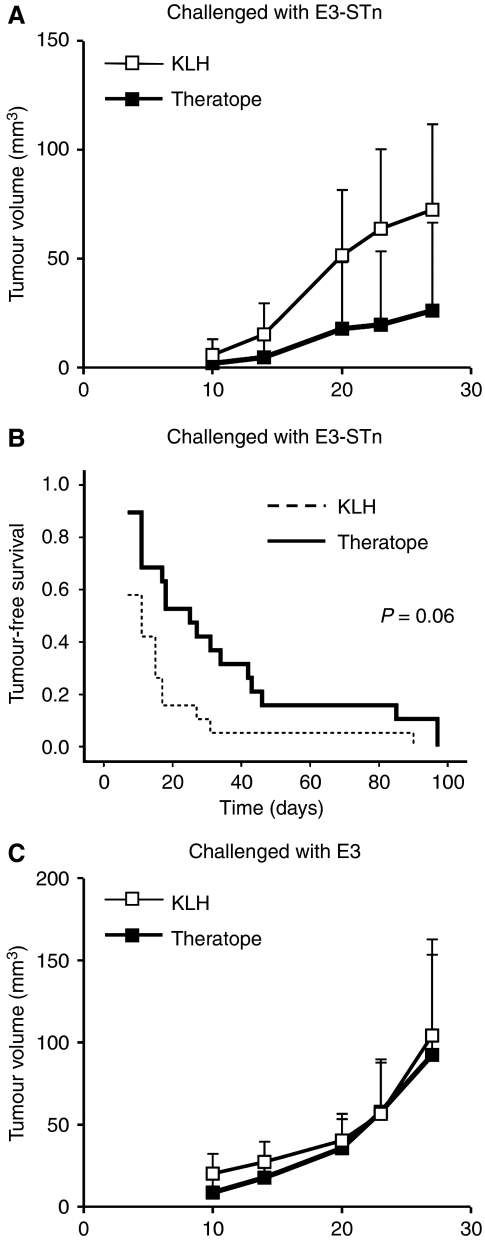
Theratope immunisation provided tumour protection. Balb/c MUC1 transgenic mice (10) were immunised with three injections Theratope or with KLH (negative control) and then tumour challenged. (**A**) Mice were challenged with the STn-positive cell line E3-STn. Growth rate was monitored by regular measurement of the tumour. (**B**) Tumour-free survival was analysed combining two independent experiments and is represented with a Kaplan–Meier graph. Statistical significance was calculated using the Breslow test. (**C**) Mice were challenged with the STn-negative cell line E3. The results in **A** and **C** are representative of two independent experiments. Bars=s.d.

Balb/c WT mice and MUC1 transgenic Balb/c mice that had been vaccinated with Theratope or controls, as described above, were tumour challenged with mammary carcinoma cell line expressing MUC1 and ST6GalNAc 1 (E3-STn) or E3 that express human MUC1 without STn. Figure 3A shows that MUC1 transgenic mice vaccination with Theratope resulted in a delay in the growth of the E3-STn tumours compared to the KLH group. Furthermore, the delay in tumour growth was associated with a significant increase in tumour-free survival as shown in Figure 3B (*P*=0.06 in the Breslow test in which a *P*-value of 0.1 is considered significant). Identical results were obtained in the WT Balb/c mice (data not shown). To confirm the importance of the STn expression in the tumour protection, we challenged Theratope-vaccinated mice with E3 tumour cells not expressing the STn glycan. In this case, no delay in tumour growth was observed, with the Theratope-vaccinated mice developing tumours as quickly as the control group (see Figure 3C) and no difference was found in the tumour-free survival (data not shown).

### The protective effect of Theratope is mediated by antibodies

**Figure 4:**
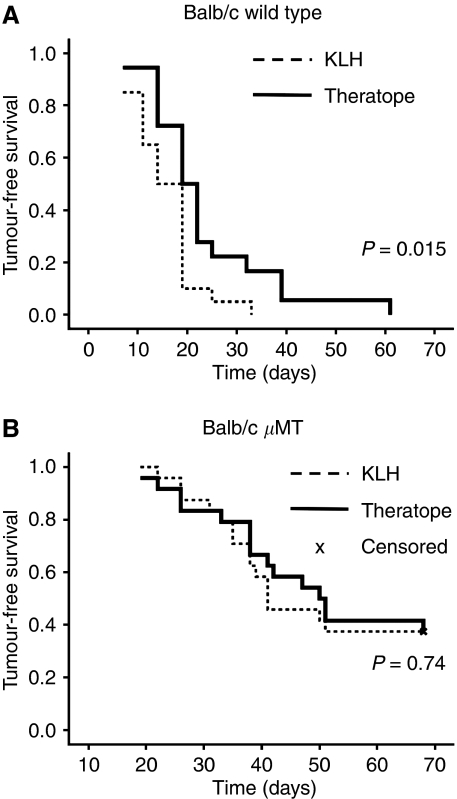
Tumour protection induced by Theratope is mediated by humoral response. Wild-type Balb/c mice (**A**) or mice deficient in antibody secretion (*μ*MT) (**B**) were immunised with Theratope or KLH (negative control). Tumour-free survival was analysed combining two independent experiments including 20 and 24 animals per group for **A** and **B** respectively and is represented with a Kaplan–Meier graph and statistical significance was calculated using the Breslow test.

To ascertain the role of anti-STn antibodies in the tumour protection observed, we immunised Balb/c *μ*MT KO mice, which are deficient in antibody production, with Theratope and tumour challenge with E3-STn cells as described above. Theratope increased tumour-free survival in WT Balb/c mice (Breslow test, *P*=0.015; Figure 4A) as described above, but not in *μ*MT mice (Figure 4B; Breslow test, *P*=0.74), demonstrating the essential role of antibodies in the tumour protection induced by Theratope immunisation.

Tumours from the mice were stained for STn expression using the mAb TKH2. Focal expression of STn was seen although all the E3-STn cells expressed STn *in vitro* (see Supplementary Figure S1). Faint but uniform staining was also detected in the extracellular compartment of all the xenografts tested. All three tumours tested from the Theratope-immunised mice showed weaker expression of STn than tumours from the mice given adjuvant and KLH alone and there appeared to be less cellular staining.

### MUC1-STn immunisation induced a humoral response but did not induce consistent protection from tumour challenge

**Figure 5:**
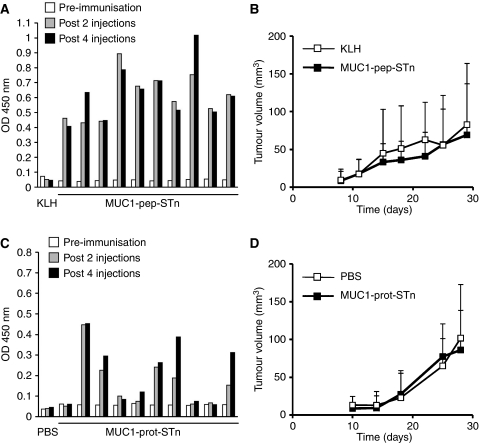
Immunisation of MUC1 TG mice with MUC1-pep-STn or MUC1-prot-STn induces anti MUC1-STn antibodies but does not provide tumour protection. Balb/c MUC1 transgenic mice (10) were immunised with MUC1-pep-STn (**A**), MUC1-prot-STn (**C**), KLH alone (control **A**) or PBS (control **C**). Sera were collected pre- and post-immunisation, and the presence of anti-STn-specific antibodies was assessed by ELISA using a MUC1-pep-STn glycopeptide-coated plate. Each set of bars represents 1 : 1000 diluted serum from a distinct animal (1 representative for the control group and 10 for the test group). OD, optical density. (**B** and **D**) After a series of four injections, mice were submitted to a tumour challenge, by injecting E3-STn cells subcutaneously. Growth rate was monitored by regular measurement of the tumour. Bars=s.d..

We have previously shown that a glycopeptide consisting of three tandem repeats of MUC1 carrying 15 STn glycans coupled to KLH can induce high titre antibodies in MUC1 transgenic mice (Sorensen *et al*, 2006; Tarp *et al*, 2007) thus breaking immune tolerance in this model. Therefore to investigate if the use of a single glycoprotein carrying STn could also induce tumour protection, we selected MUC1-carrying STn as an immunogen. Balb/c MUC1 transgenic mice were immunised MUC1-pep-STn coupled to KLH (Sorensen *et al*, 2006; Tarp *et al*, 2007) or MUC1-prot-STn (see Materials and Methods). All the mice vaccinated with MUC1-pep-STn developed high titre antibodies reactive with MUC1-STn (Figure 5A). In contrast to the antibodies raised against Theratope, the antibodies cross-reacted with MUC1-Tn (Supplementary Figure S2A) and stained primary breast tumours expressing STn and Tn (data not shown). Only 50% of the mice vaccinated with MUC1-prot-STn developed antibodies reactive with MUC1-STn (Figure 5C). These sera did not react with unglycosylated MUC1 peptide and, unlike the antibodies induces to MUC1-pep-STn, did not cross-reacted with the MUC1-Tn glycoform (Supplementary Figure S2B). Antibodies induced to MUC1-pep-STn and MUC1-prot-STn were mostly of the IgG1 subtype but mice immunised with MUC1-pep-STn also developed IgG2a-specific antibodies (see Supplementary Figure S3). Using Cos7 cells transfected with MUC1 and/or ST6GalNAc I, we showed that anti-sera from immunised mice only reacted with Cos7 expressing both MUC1 and STn (see Supplementary Figure S2C). Moreover, these sera reacted with only with primary human breast cancers that expressed MUC1 and STn (data not shown).

The MUC1-pep-STn- or MUC1-prot-STn-vaccinated mice were tumour challenged with E3-STn cells. Although the initial experiment using MUC1-pep-STn gave a delay in the growth of tumours in the vaccinated mice compared to the KLH control, this was not repeated in two subsequent experiments where no difference was observed in tumour growth between vaccinated and control animals (Figure 5B). In addition, when MUC1-prot-STn was used as the immunogen, no difference was seen in the growth of vaccinated mice compared to control groups in any experiment (Figure 5D). Injection of cyclophosphamide 3 days before MUC1-pep-STn or MUC1-prot-STn vaccination had no effect on the rate of tumour growth and efficacy of tumour protection (data not shown).

Thus, 10 *μ*g per injection of MUC1-pep-STn or MUC1-prot-STn could induce humoral responses in MUC1 transgenic mice but at the concentration and in the model used here this did not result in consistent protection from tumour challenge.

### Identification of glycoproteins carrying STn antigen

**Figure 6:**
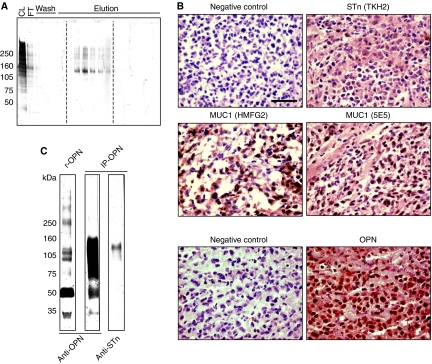
OPN is an STn-positive protein. (**A**) Fractions from a TKH2 affinity column (Elution) were subjected to western blotting with TKH2 after being separated on a 4–15% gradient SDS–PAGE. CL, cell lysate; FT, flow through. (**B**) Frozen sections of murine tumours from E3STn were stained with TKH2 to detect STn, with HMFG2 and 5E5 to detect MUC1 or a specific polyclonal goat IgG to detect OPN. Appropriate negative controls were obtained by incubating the sections with a non-relevant primary antibody. Bar=10 *μ*m. (**C**) OPN was immunoprecipitated from murine tumour lysates (IP OPN), analysed by SDS–PAGE and western blotted with anti-OPN and -STn as indicated. Western blot analysis of recombinant OPN (r-OPN) is shown for comparison.

Because only anti-sera recognising several STn-carrying proteins were able to mediate tumour protection in our model, it was of interest to identify these proteins to understand the effect of Theratope immunisation. We used TKH2 mAb to isolate STn-proteins from E3-STn cell lystates by immuno-affinity chromatography. Fractions were analysed by western blot using TKH2 mAb (Figure 6A). Sialyl-Tn-positive fractions were pooled, submitted to further SDS–PAGE and 22 individual silver stained bands ranging from 50 to >250 kDa were analysed by tandem mass spectrometry analysis after in gel trypsin digestion. Most of the candidates identified appeared to be abundant cytosolic contaminant proteins (actins, myosin 9, heat shock proteins) (see Supplementary Table S1). However, one candidate identified from a 50 kDa band, the precursor of OPN, appeared to be of interest. Osteopontin is an extracellular matrix protein known to be involved in a number of physiological and pathological events, such as cell-mediated immune response, inflammation, cell survival and cancer metastasis (Denhardt *et al*, 2001). Human OPN has a cluster of five *O*-glycosylated threonines located next to an RGD integrin-binding site domain (Christensen *et al*, 2005). Three of these threonines are strictly conserved in mammals and OPN has been found glycosylated in mouse (Christensen *et al*, 2007).

Therefore, we analysed the E3-STn tumours developed in the mice for OPN and STn expression. Immunohistochemistry revealed the homogeneous presence of both OPN and STn in the tumour (Figure 6B). Osteopontin was homogenously expressed in the tumour, similar to MUC1 as detected with HMFG2 or 5E5 antibody (Figure 6B). To confirm the presence of STn as *O*-glycan linked to OPN, we immunoprecipitated OPN from whole tumour lysates and western blotted with TKH2 mAb (Figure 6C). Recombinant unglycosylated OPN was also probed with an anti-OPN antibody as control for the electrophoresis pattern. The blot showed that the major form enriched from the tumour was a high molecular form of OPN detected as a smear, indicative of various degrees of post-translational modification. A small portion of this immunoprecipitated OPN was detected by TKH2, indicating that at least part of the OPN found in the tumour was bearing STn determinants.

## Discussion

Glycosylation is one of the most common forms of post-translational modifications and is essential for many protein and cellular functions. In malignant cells, changes in carbohydrates attached to proteins and lipids are frequently observed, resulting in the expression of cancer-associated glycans. One such glycan, STn, is found on 25–30% of breast carcinomas but its expression by normal tissues is highly restricted, making it an attractive therapeutic target. However, it is generally thought that for active immunotherapy to be effective it is necessary to induce effector CD8+ T cells and as unconjugated glycans cannot induce T-cell responses, their use as immunogens has been restricted. However, MUC1-carrying STn has been shown to induce high titre antibodies in MUC1 transgenic mice (Sorensen *et al*, 2006; Tarp *et al*, 2007).

In this paper we report the use of three different STn-based immunogens, that are synthetic STn coupled to KLH (Theratope), MUC1-pep-STn coupled to KLH and MUC1-prot-STn, in a MUC1 transgenic mouse model to induce tumour protection. Despite all the immunised mice developing a strong humoral response we did not observe consistent tumour protection when the mice were vaccinated with MUC1-pep-STn or MUC1-prot-STn. However, further optimisation of the dose and delivery method may enhance the efficacy of the glycopeptide and glycoprotein used as immunogens. In contrast, immunisation with Theratope, thus making the immunogen independent of a specific peptide backbone, did result in a significant delay in tumour development. Furthermore, this effect was strictly dependant on STn being present in the tumour cells.

The Theratope-immunised mice developed antibodies that recognised STn carried on MUC1, but also on several other proteins expressed by E3-STn cells or other STn-positive cell lines. Tumour challenge experiments performed in Balb/c *μ*MT mice, which cannot produce immunoglobulins showed no delay in tumour growth after vaccination with Theratope, definitively proving that the tumour protection was dependant upon anti-STn antibodies. It can be noted that tumour appearance in *μ*MT mice is delayed compared to WT mice. This phenomenon has previously been documented by Shah *et al* (2005) who showed that mice genetically lacking B cells were more resistant to tumour by loss of B-cell inhibition of the anti-tumour T cells.

Antibodies could delay tumour growth by antibody-dependent cellular cytotoxicity, inhibition of function or a combination of the two mechanisms, and indeed Theratope induced antibodies of the IgG2a subtype that mediate ADCC in mice (Matthews *et al*, 1981; Herlyn and Koprowski, 1982). However, MUC1-pep-STn also induced IgG2a antibodies (see Supplementary Figure S3) but no tumour protection was observed in mice immunised with this immunogen. To determine if inhibition of function is involved in the delayed tumour growth reported here, it is first necessary to identify target glycoproteins. Surprisingly, although cancer cell lines seem to express a variety of *O-*glycoproteins carrying STn epitope, few of these proteins have been identified (Clement *et al*, 2004; Julien *et al*, 2006). Using affinity chromatography and mass spectrometry, we identified OPN as an STn-positive protein that was expressed by the tumour. This is the first time a secreted protein of the extracellular matrix has been shown to be modified by cancer-associated glycosylation.

Osteopontin is expressed in most normal tissues during remodelling. It functions both as a cell attachment and chemotactic protein, mainly by interacting with integrins through an RGD domain, and also by interacting with CD44 (Denhardt *et al*, 2001). Interestingly, sialylation of OPN has been shown to influence its binding properties (Shanmugam *et al*, 1997). Because OPN is expressed in most primary carcinomas (Brown *et al*, 1994) it is thought to have a basic function in tumour progression, and in breast cancer over-expression of OPN is associated with lymph node metastasis and poor prognosis (see Tuck *et al*, 2007 for review). Proposed mechanisms of action of OPN include induction of survival, enhanced migration or immune regulation. Thus antibodies that bind STn carried on OPN produced by the tumour cells may block the function of this glycoprotein leading to a delayed tumour growth. Our initial results suggest that further studies into targeting specific glycoforms of OPN as a potential therapeutic approach in STn-positive breast cancers is warranted.

The results presented in this study demonstrate that STn glycans as present in Theratope can effectively produce tumour protection against STn-expressing cancer cells, which is strictly dependant on the induction of antibodies reactive with STn. The results also suggest that targeting STn carried on several proteins rather than only one is more likely to interfere with various mechanisms involved in tumour development. Moreover the targeting of multiple antigens may be advantageous in overcoming the problem of immunoediting that has been documented in a number of clinical studies (Dudley *et al*, 2005; Singh and Paterson, 2007) and although diminished STn expression could still be found on tumours from mice vaccinated with Theratope. In the light of our results, we suggest that anticancer strategies using Th2 immune response have been underestimated and warrant further investigations. Moreover, the expression of STn on the tumour is crucial for an effect on tumour growth. In the phase III clinical trial with Theratope where significant therapeutic benefit was not observed, the STn expression of the tumours was not documented. Thus it is possible that the subgroup of patients with STn-expressing tumours may have benefited but the effect was obscured by the enrolment of patients whose tumours did not express the glycan.
