# Supplementary material for: Sialyl-Tn vaccine induces antibody-mediated tumour protection in a relevant murine model
Source: Br J Cancer. 2009 May 12;100(11):1746–54. doi: 10.1038/sj.bjc.6605083 (PMC2695689; doi:10.1038/sj.bjc.6605083)
Supplement: Supplementary Information [file 6605083x4.doc]

**Supplementary Data**

**Material and methods**

**COS7 transfection**

Cos cells were transiently transfected with ST6GalNAc-1 and/or MUC1 in pcDNA3.1 using Fugene 6 transfection reagent (Roche). Forty-eight hours later the cells were fixed with methanol/acetone (1:1) and stained with HMFG2 for MUC1 expression, TKH2 for STn expression and with the antiserum from MUC1-prot-STn immunised mice. Pre-immune serum was used as a negative control. Binding was detected using rabbit anti-mouse IgG (Dako, Denmark) and the staining visualised using a Zeiss Axiofluor fluorescence microscope.

**Figure legends:**

Figure S1: **Tumours grown in mice express STn.** Paraffin embedded sections of tumours obtained from mice immunised as indicated were stained for STn expression using TKH2 mAb followed by biotinylated anti-mouse Ig and StreptABcomplex/HRT. CTRL, sections incubated with buffer instead of TKH2.

Bars =50µM.

Figure S2: **Tn Cross-reactivity in MUC1-STn immunisation**. A: 10 Balb/c MUC1 transgenic mice were immunised with MUC1-pep-STn or injected with KLH (negative control). Sera were collected pre- or post-immunisation and the presence of anti-Tn specific antibodies was assessed by ELISA using a MUC1-pep-Tn glycopeptide-coated plate. B: the Same experiment was performed using mice immunised with MUC1-prot-STn or PBS (negative control). Each set of bars represents a distinct animal (1 representative for the control group and 10 for the test group). O.D.: optical density. C: COS-7 cells were transfected with either human ST6GalNAc I (top row), human MUC1 (middle row) or co-transfected with both (bottom row). Staining was performed using monoclonal antibodies specific to STn (TKH2) and MUC1 (HMFG2) or antiserum from immunised mice. This picture is representative of 3 independent experiments. Bar: 10 μm.

Figure S3: **Isotype of anti-STn antibodies** raised after immunisation by Theratope® (triangle), MUC1-pep-STn (diamond) or MUC1-prot-STn (circle) was analysed by ELISA in wells coated with 200ng of OSM (Theratope® group) or 50ng of MUC1-pep-STn (other groups), using isotype specific secondary antibody for detection. Sera were used diluted 1:3000 for Theratope® group and 1:1000 for the others. Each dot is a distinct animal and bars represent the average measured O.D. O.D.: optical density.

**Table S1: Identified proteins in the 50 kDa-SDS-PAGE band. UniProt accession number.**

| **Protein** | **Name of protein** | **Theoritical MW (Da)** | **accession number** | **Mascot Score** | **Number of peptides** | **% covered** |
| --- | --- | --- | --- | --- | --- | --- |
| ACTB | Actin, cytoplasmic 1 | 42052 | Q6ZWM3 | 176 | 9 | 23 |
| ACTA | Actin, aortic smooth muscle | 42381 | P62737 | 129 | 6 | 24 |
| ODP2 | Dihydrolipoyllysine-residue acetyltransferase component of pyruvate dehydrogenase complex | 68469 | Q8BMF4 | 83 | 1 | 3 |
| K2C1 | Keratin, type II cytoskeletal 1 | 66079 | P04104 | 81 | 1 | 1 |
| LMNA | Lamin-A/C | 74478 | Q9DC21 | 57 | 1 | 1 |
| OSTP | Osteopontin precursor | 32554 | P10923 | 35 | 1 | 8 |
